# Current Opinion on Diagnosis of Peripheral Artery Disease in Diabetic Patients

**DOI:** 10.3390/medicina60071179

**Published:** 2024-07-20

**Authors:** Francesca Ghirardini, Romeo Martini

**Affiliations:** Department of Angiology, San Martino Hospital, 32100 Belluno, Italy; angiologia.bl@aulss1.veneto.it

**Keywords:** peripheral arterial disease, diabetes mellitus, diagnostic test, CLI, CLTI

## Abstract

Peripheral arterial disease (PAD) prevalence and diabetes mellitus (DM) prevalence are continuously increasing worldwide. The strong relationship between DM and PAD is highlighted by recent evidence. PAD diagnosis in diabetic patients is very important, particularly in patients with diabetic foot disease (DFD); however, it is often made difficult by the characteristics of such diseases. Diagnosing PAD makes it possible to identify patients at a very high cardiovascular risk who require intensive treatment in terms of risk factor modification and medical therapy. The purpose of this review is to discuss the diagnostic methods that allow for a diagnosis of PAD in diabetic patients. Non-invasive tests that address PAD diagnosis will be discussed, such as the ankle-brachial index (ABI), toe pressure (TP), and transcutaneous oxygen pressure (TcPO2). Furthermore, imaging methods, such as duplex ultrasound (DUS), computed tomography angiography (CTA), magnetic resonance angiography (MRA), and digital subtraction angiography (DSA), are described because they allow for diagnosing the anatomical localization and severity of artery stenosis or occlusion in PAD. Non-invasive tests will also be discussed in terms of their ability to assess foot perfusion. Foot perfusion assessment is crucial in the diagnosis of critical limb ischemia (CLI), the most advanced PAD stage, particularly in DFD patients. The impacts of PAD diagnosis and CLI identification in diabetic patients are clinically relevant to prevent amputation and mortality.

## 1. Introduction

Peripheral arterial disease (PAD) is mainly caused by atherosclerotic stenosis or obstruction of the lower limb arteries.

Several epidemiologic observations have addressed the continuously increasing prevalence of PAD worldwide [1]. The aging of the population and the increased incidence of diabetes mellitus (DM) [2] seem to be the main risk factors involved in this trend. 

PAD affects 20–28% of the diabetic population, and up to 50% of patients with diabetic foot disease (DFD) [3,4]. Racial and socioeconomic factors may also significantly affect the epidemiological data of PAD in this group of patients, as diabetes mellitus accelerates the disease progression and worsens the severity of PAD [5,6].

Despite PAD often being asymptomatic, it can cause intermittent claudication or critical limb ischemia (CLI) with a poor quality of life, rest pain, non-healing skin ulcers, or gangrene, which may lead to limb amputation [7]. PAD patients have also a high burden for cardiovascular and all-cause mortality rates [8].

The classical symptoms of PAD, such as intermittent claudication or rest pain, may be reduced or totally absent in diabetic patients because of their sedentary lifestyle and a reduced sensation of pain related to peripheral neuropathy, complicating the clinical diagnosis of PAD in diabetic patients [9].

Moreover, co-existent medial artery calcification can affect the accuracy of non-invasive diagnostic tests such as the ankle-brachial index (ABI) by causing an elevation in ankle pressure [10,11].

Consequently, diabetic PAD patients remain underdiagnosed, as well as being at a higher risk for major adverse limb events (MALEs) and major adverse cardiac events (MACEs) [12] with a higher morbidity, amputation rate, and mortality [13,14].

This brief review aims to focus on the current opinion on the diagnosis of PAD in diabetic patients, hoping to improve interest and attention about this topic, which may be considered as one of the main forthcoming problems in vascular disease management and general health.

## 2. Material and Methods

This review was performed through searches in the PubMed, Scopus, Medline, and NCBI databases.

The terms used were “peripheral artery disease”, “critical limb ischemia”, “chronic limb threatening ischemia”, “diagnosis”, “diagnostic test”, “imaging”, “ankle-brachial index”, “toe pressure”, “toe-brachial index”, “transcutaneous oxygen pressure”, “duplex ultrasound”, “computed tomography angiography”, “magnetic resonance angiography”, “digital subtraction angiography”, “diabetes”, “diabetic patients”, “diabetic foot”, “diabetic foot”, and “diabetic foot complications”.

To obtain a more comprehensive vision and systematic review, consensus documents and guidelines reported by leading scientific societies were consulted from 1990.

## 3. Diagnosis of PAD in Diabetic Patients

All the consulted documents reported that PAD should be suspected in diabetic patients at a high cardiovascular risk < 65 years, in those with other atherosclerotic diseases (coronary disease, carotid disease, etc.), in those with other conditions (abdominal aortic aneurysm, chronic kidney disease, heart failure, etc.), and in those with non-healing lower extremity wounds, in particular [15,16,17].

### 3.1. Clinical History and Physical Examination

Most documents strongly recommend reviewing patient clinical history and performing a physical examination with vascular auscultation and pulse palpation, in addition to skin inspection [7,15,16,18,19].

The most common symptom of PAD is intermittent claudication (painful muscle cramping in the hips, thighs, or calves when walking). Rest pain may occur in advanced stages of the disease.

The Leriche Fontaine [20] and Rutherford [21] classifications are based on clinical symptomatology (Table 1).

Peripheral neuropathy may negatively influence the use of these classifications in selecting the severity of PAD in diabetic patients.

The most typical examination sign regarding PAD is the absence of a pulse.

Also, skin that is dry, flaky, shiny, or cracked, abnormal nail growth, a loss of leg hair, cold foot, and atrophic muscles are signs of a reduced blood supply.

Clinical examinations should be performed on diabetic patients [17], but they are not highly reliable for diagnosing or excluding the disease. In fact, studies have shown that the presence of a pulse does not exclude PAD [22,23,24,25].

Moreover, pulse palpation may be difficult for the presence of peripheral edema related to renal failure or autonomic neuropathy.

### 3.2. First-Level Non-Invasive Diagnostic Tests: Ankle Pressure and Ankle-Brachial Index (ABI)

Systolic ankle pressure (AP) is measured at rest in the supine position by a CW Doppler, photoplethysmography, or laser Doppler probe, positioning a pneumatic cuff at the ankle.

The ankle-brachial index is the ratio of the pressure measured at the ankle to the systolic arterial pressure of the brachial artery.

The normal values of the ABI are in the range of >0.90 <1.30–1.40.

Patients with an ABI of ≤0.90 are diagnosed with PAD. Also, AP is reported in guidelines as a diagnostic criterion for PAD, particularly for CLI [15,19,26,27,28,29].

The ABI is a simple, inexpensive, quick test, even if it does not provide disease localization.

In non-diabetic patients, the ABI has a sensitivity between 68% and 84% and a specificity from 84% to 99% [30,31,32,33].

The ABI is a predictor of disease severity: the lower the ABI, the stronger the impact of atherosclerotic disease for the patient [34]. In addition, the ABI provides data to predict wound healing and limb survival, and it is useful for monitoring the efficacy of therapeutic intervention [35].

However, medial artery calcification can negatively affect the accuracy of AP and the ABI by causing a false elevation in ankle pressure [36]. An ABI of >1.30–1.40 may mask artery stenosis with an increase in both cardiovascular and mortality risk [37].

Several studies in the diabetic population [22,24,25,38,39,40,41,42,43,44,45,46,47,48,49,50,51,52,53,54,55,56,57,58,59,60,61,62,63,64,65,66,67,68,69,70] have shown that an ABI of <0.9 is associated with a moderate–large likelihood of PAD, with a positive likelihood ratio of 2.1–19.9 and a negative likelihood ratio of 0.29–0.84 in patients without DFD and a positive likelihood ratio of 1.69–2.32 and a negative likelihood ratio of 0.53–0.75 in patients without DFD [71].

### 3.3. Second-Level Non-Invasive Diagnostic Tests

#### 3.3.1. Toe Pressure and Toe-Brachial Index (TBI)

The measurement of systolic toe pressure (TP) is performed at rest in the supine position by a digital pneumatic cuff placed at the base of the big toe and photoplethysmography and a laser Doppler probe at the tip of the toe.

The toe-brachial index (TBI) is the ratio between TP and brachial systolic arterial pressure.

Toe pressure is generally considered to be 6–10 mmHg less than brachial pressure, due to the distance from the heart and the smaller size of the digital arteries. A toe pressure of ≤50 mmHg is generally indicative of reduced perfusion, and ≤30 mmHg is indicative of CLI [19,28], while the TBI is considered to be pathological if it is ≤0.7 [7,15,16,18,35,72].

TP and TBI are simple, inexpensive, and quick tests.

Some studies on diabetic patients [23,24,25,38,42,47,59,64] have shown that a TBI of <0.7 has a moderate ability to diagnose and exclude PAD with a positive likelihood ratio of 2.0–3.55 and a negative likelihood ratio of 0.25–0.44 [71].

TP and TBI overcome the limitation of the ABI in diabetic patients, as the calcification of the artery wall is less present in the foot arteries than in the legs.

They can provide data to predict wound healing and limb survival, and are useful in follow-ups after revascularization [35].

An important and frequent negative issue is that TP and TBI cannot be measured after toe amputation.

#### 3.3.2. TcPO2

Transcutaneous oxygen pressure (TcPO2) allows for the detection of the blood partial pressure of oxygen in a non-invasive way by a probe with a Clark electrode heated to 44 °C and applied to the skin.

TcPO2 measurement requires preliminary calibration of the electrode with air. The room temperature must be in the range between 22 and 24 °C.

Where to place the probe on the skin should be carefully chosen to avoid damaging the skin area because of the 44 °C temperature.

Normal TcPO2 values range between 60 and 70 mmHg in ambient air.

TcPO2 seems to add no important information on asymptomatic or claudicant patients, but it has a clinical impact in the case of critical ischemia, initially diagnosed when TcPO2 is <10 mmHg in the supine position and <45 mmHg in the decline position [73].

Values of <30 mmHg indicate critical skin perfusion and are used to select CLI [7,15,16,17,18,19,27,35,72].

In fact, TcPO2 of < 30 mmHg is an independent predictor of lesion non-healing [74] and correlates with the risk of amputation [75].

In diabetic patients, it can be used to predict the possibility of healing DFD [76,77,78,79,80,81,82,83,84,85].

However, TcPO2 measurement may have a limited accuracy in the presence of edema or infection.

#### 3.3.3. Pedal Doppler Waveforms

Continuous wave Doppler ultrasound (CWD) is frequently used as a non-invasive limb vascular assessment for PAD diagnosis, because it is a low-cost screening test that is accessible and quick to use [62]. Monophasic Doppler waveforms are diagnostic for PAD. However, the interpretation of CWD waveforms is operator-dependent, and considered to be more subjective than pressure measurements.

In DM patients, a preliminary study demonstrated that CWD had a higher sensitivity and specificity for PAD diagnosis than the ABI or TBI [23]. This seems to be confirmed by a recent meta-analysis showing that the presence of a visual monophasic pedal Doppler waveform has the ability to diagnose and exclude PAD with a positive likelihood ratio of 7.09 and a negative likelihood ratio of 0.19 [71].

### 3.4. Non-Invasive and Invasive Imaging Methods

Adequate imaging is indicated to diagnose the anatomical localization and severity of artery stenosis or occlusion in PAD [7,15,16,17,18,35,72].

Duplex ultrasound (DUS), computed tomography angiography (CTA), magnetic resonance angiography (MRA), and digital subtraction angiography (DSA) are useful imaging techniques that allow for obtaining anatomical information on the disease.

Each of these techniques has its own advantages and disadvantages, and the choice of method is made considering the patient’s clinical condition and the availability of equipment and local expertise.

#### 3.4.1. Duplex Ultrasound

DUS is indicated as the first diagnostic method in PAD to enable the visualization and localization of vascular stenoses and occlusions [7].

DUS is a non-invasive, safe, reliable, repeatable, low-cost technique that can be performed at a patient’s bedside, does not require patient cooperation, and does not use ionizing radiation, however, it is operator-dependent and visualization is sometimes difficult, particularly in the case of calcification or structures close to bone or gas-filled cavities [86,87,88,89].

Particularly in diabetic patients, the presence of medial arterial calcification makes it difficult to study leg vessels with DUS.

Additional imaging techniques are recommended if DUS is not accurate enough in describing the disease and if proceeding to elective surgical revascularization.

#### 3.4.2. Computed Tomography Angiography

CTA is a rapid diagnostic investigation with a good accuracy; however, it uses ionizing radiation and iodine contrast, so there is a relative contraindication in the case of allergic diathesis or CKD. Again, the presence of calcification can compromise imaging accuracy [90,91,92].

#### 3.4.3. Magnetic Resonance Angiography

MRA is a non-invasive imaging technique with high rates of sensitivity and specificity which uses neither ionizing radiation nor iodine contrast, but gadolinium contrast medium. However, it is a method that requires longer image acquisition times, is not available in all centers, and cannot be used in patients with incompatible ferromagnetic devices [88,93,94].

#### 3.4.4. Digital Subtraction Angiography

DSA is the gold standard in vascular imaging for PAD, particularly in patients suspected of CLTI [7,35,95]. DSA is an invasive technique that can cause complications (bleeding, hematoma, false aneurysm, arteriovenous fistula, and contrast-related complications), but it should minimize both the use of iodine contrast and the dosage of ionizing radiation while maximizing distal vasculature imaging. Its great advantage is that it is diagnostic and can be interventional at the same time [96,97,98,99].

## 4. Foot Perfusion Assessment for Diagnosis of CLI

Critical limb ischemia (CLI) is the most advanced PAD stage, burdened by the highest rates of limb amputation and cardiovascular mortality. DM patients with CLI had a significantly increased risk of death compared to non-diabetics, suggesting that PAD patient mortality strongly depends on whether the PAD patient suffers from DM [100].

Therefore, the diagnosis of PAD, and more specifically of CLI, in DFD becomes extremely important, as it allows for the identification of diabetic patients at a higher risk of amputation and cardiovascular or all-cause mortality, so they can be quickly referred for revascularization attempt when possible.

The non-invasive tests described address PAD diagnosis, but also allow for assessing foot perfusion. The ability of these tests to provide a hemodynamic assessment of limb perfusion makes these tests very useful in diagnosing CLI (Table 2).

Considering the clinical definition of CLI, first proposed in 1982, later consensus and guidelines tried to provide hemodynamic parameters that would allow for a more objective definition of the condition.

In 1987, the European Working Group first reported an AP of ≤50 mmHg as a parameter for CLI diagnosis [26]. In 1989, a second paper by the same working group provided a definition of CLI in patients with and without diabetes, introducing a TP of ≤30 mmHg as an alternative to AP [27].

Rutherford, in the 1997 classification, introduced an AP of <40 mmHg or a TP < of 30 mmHg for class 4, and an AP of <60 mmHg or a TP of <40 mmHg for classes 5 and 6 [21].

In 2000 and in 2007, the Trans-Atlantic Inter-Society Consensus Document on Management of Peripheral Arterial Disease introduced the parameter of TcPO2 for CLI diagnosis, with cut-off of 50 mmHg in the presence of wounds or gangrene and 30 mmHg in the presence of rest pain, in addition to an AP of <70–50 mmHg and a TP of <50–30 mmHg [19,28].

In 2011, the European Society of Vascular and Endovascular Surgery reiterated that AP and ABI are not reliable parameters for CLI diagnosis, and it recommended measuring the TP in all patients suspected of CLI and stratifying the risk of amputation through TcPO2 measurement [101].

As can be observed, for years, international guidelines have based the definition of CLI on the establishment of hemodynamic cut-offs of ischemia with ABI, SP, TP, TBI, and TcPO2.

However, changes in the pattern of PAD presentation, caused by diabetes and renal failure, have made the hemodynamic criterion alone insufficient to include all patients at a very high risk of limb loss and mortality. In particular, medial sclerosis is a major risk factor for CLI, failure of endovascular treatment, amputation, and mortality [102].

Furthermore, the ischemia parameter alone does not allow for including the heterogeneous spectrum of DFD patients who may have a moderate or subcritical reduction in foot perfusion but an evidently high clinical risk because of the presence of large and/or infected lesions [103].

The breakthrough in the redefinition of CLI patients at a higher risk of amputation came with the Lower Extremity Threatened Limb Classification System proposed by the Society of Vascular Surgery in 2014 based on Wound, Ischemia, and foot Infection (WIfI) [29].

For the first time, the classification system combined the hemodynamic parameters that quantify the perfusion deficit (ischemia) with the assessment of the wound extent and the infection presence and severity. This new system allows for including the heterogeneous populations previously excluded from the CLI criteria, with a more realistic prediction of patients at a risk of amputation and candidates for revascularization.

The WIfI classification had profound repercussions for the nosographic definition of critical ischemia, up to the proposal of the term Chronic Limb-Threatening Ischemia (CLTI) to replace CLI.

This new definition was included in the 2017 ESC guidelines for the diagnosis and treatment of PAD [15], while, until a year earlier, only CLI was mentioned in the AHA/ACC guidelines [72].

In 2019, for the first time, guidelines for the treatment of CLTI were formulated by a collaboration between the Society for Vascular Surgery, the European Society for Vascular Surgery, and the World Federation of Vascular Societies; in those guidelines, the WIfI classification was accepted for the definition of patients at a high risk of limb loss [35].

Even if the 2019 guidelines of the European Society of Vascular Medicine still report the term CLI, based on the Rutherford classification, despite inserting the WIfI classification for classifying the infection [7], the new ACC/AHA guidelines embrace the new terminology [16].

## 5. Discussion

PAD is a global burden, but it remains frequently underestimated [1]. PAD diagnosis makes it possible to identify populations of patients at a very high cardiovascular risk, for whom it is necessary to provide the best medical treatment for reducing morbidity and mortality.

In the 2017 ESC guidelines and the most recent ACC/AHA guidelines, screening with ABI is recommended in men and women aged <65 years and classified at a high cardiovascular risk according to the ESC guidelines, which also include DM patients (except for young people with type 1 diabetes without other major risk factors) [15,16].

There is a strong relationship between DM and PAD [3].

Early PAD diagnosis in diabetic patients is particularly important for preventing later complications of the disease, such as non-healing ulcers or gangrene, and for reducing major adverse limb events, major adverse cardiovascular events, and mortality.

However, PAD diagnosis in diabetic patients is often made difficult by the characteristics of the diseases.

Characteristic PAD symptoms are frequently absent in diabetic patients. Often, patients do not report claudication due to a lack of physical activity, and do not refer pain due to concomitant neuropathy [104]. This implies that PAD diagnosis is made already at the most advanced stages of the disease, often when patients already present DFD.

Guidelines recommend PAD screening in diabetic patients at a high cardiovascular risk aged <65 years, and particularly in DFD [15,16,17]. But in clinical practice, this rarely occurs.

Collecting clinical history and examining the feet for signs of ischemia and palpation foot pulse are recommended, but are not able to diagnose or exclude PAD alone.

In fact, the presence of a pulse does not exclude the disease [22,23,24,25], and some conditions (e.g., edema) can make it difficult to palpate pulses.

So, additional tests can be used to diagnose PAD.

The first-level non-invasive diagnostic test is ABI. An ABI of ≤ 0.90 is diagnostic for PAD and is recommended as the first-line test by the guidelines [6,7,15,16,17,18,35,72].

However, some features of PAD in diabetic patients, such as the distal location of the disease (infrapopliteal) [105,106] and the pattern of medial arterial calcification [36], frequently make ABI unreliable. In fact, in patients with falsely elevated ABI (>1.3–1.4), an increase in cardiovascular and mortality risk is observed [37].

For this reason, in diabetic patients, second-level non-invasive diagnostic tests are suggested.

Toe pressure (TP) and the toe-brachial index (TBI) are valid alternatives for PAD diagnosis [71]. A TBI of ≤0.7 is diagnostic for PAD. A TP of ≤50 mmHg is generally indicative of reduced perfusion, while ≤30 mmHg is indicative of CLI.

TP and the TBI are useful in the presence of non-compressible tibial arteries, because there is less calcification of the vessels in the foot arteries. So, these tests are particularly important in diabetic patients [64]. However, the measurement requires the presence of toes, so it is not feasible in patients who undergo proximal or distal amputations.

Monophasic Doppler waveforms are considered to be diagnostic for PAD [71], even if they are operator-dependent [23].

If PAD is suspected in a diabetic patient, a multi-test approach was recently proposed by the intersocietal IWGDF, ESVS, and SVS guidelines [17]. Performing the ABI and TBI in combination with pedal Doppler waveforms is suggested for diabetic people without foot ulcers.

The guidelines underline that there are no values of the ABI, TBI, or Doppler waveforms above which PAD can be excluded, but PAD is less likely in the presence of an ABI of 0.9–1.3, TBI of ≥0.70, and triphasic or biphasic pedal Doppler waveforms [17]. For this reason, if clinical suspicion is high, it is reasonable to consider researching PAD through imaging methods.

In DFD patients, the diagnostic approach is the same; however, if toe pressure cannot be measured, TcPO2 measurement is suggested [17].

TcPO2 is the only microcirculatory test that allows for obtaining information about skin perfusion [74]. This is particularly important in diabetic patients in whom arterial wall calcification and/or finger amputation make it impossible to apply other methods of evaluation.

TcPO2 is a predictor of lesion non-healing [74], and this is particularly important in DFD [76,77,78,79,80,81,82,83,84,85]. TcPO2 also correlates with the risk of amputation [75].

A TcPO2 of <30 mmHg was assumed in the WIfI classification to define the degree of severe ischemia (grade 3), while TcPO2 values between 30 and 39 mmHg define grade 2 ischemia [29]. However, TcPO2 is included among the criteria for CLI diagnosis in the guidelines [7,15,16,17,18,19,27,35,72] and is also reported in the CLTI management guidelines [29].

To obtain the anatomical localization and severity of artery stenosis or occlusion in PAD, it is necessary to refer to imaging methods.

DUS is the first diagnostic method in PAD because it is a non-invasive, low-cost technique, however, it is operator-dependent and visualization is sometimes difficult [86,87,88,89].

DSA is the gold standard in imaging methods for PAD [7,35,95], but it is an invasive technique.

Imaging assessments in revascularization strategy planning are based on anatomical staging provided by the TASC classification for aorto-iliac and femoral-popliteal disease and the recent Global Anatomic Staging System (GLASS) for infrainguinal and pedal disease [19,35].

In clinical practice, CTA is the most used technique to study large vessels for surgical and endovascular intervention planning. DSA is mostly used for the study and treatment of distal vessels when large vessels have no significant pathology, because it is diagnostic and interventional at the same time.

In diabetic patients, the utility of DUS and CTA for PAD diagnosis can be affected because of the frequent distal localization of the disease and the presence of medial arterial calcification in the leg vessels, and DSA may be preferable, but there is not enough evidence about that yet.

In diabetic patients, particularly those with DFD, the stratification of amputation risk is fundamental in selecting patients eligible for revascularization.

The multiparametric evaluation proposed in the WIfI classification for limb amputation risk assessment appears to be particularly suitable for diabetic patients. In fact, in DFD patients, wound healing is not only related to the degree of ischemia, but also to the lesion extent and the possible concomitant presence of infection.

The early identification of diabetic patients at a higher risk of amputation allows them to be quickly referred to imaging tests that may confirm PAD, and to a possible attempt at early revascularization that may allow for salvaging limbs.

The new definition of CLTI is particularly important for diabetic patients with PAD because it allows patients with DFD who may have moderate ischemia and are still at risk of limb loss to be referred for imaging and revascularization.

## 6. Conclusions

The early detection of PAD in diabetic patients is very important to prevent worse outcomes. However, diagnosis is made difficult by the peculiar features of PAD in diabetes that can mask the symptoms and signs and reduce the reliability of diagnostic tests. Therefore, PAD in diabetic patients should always be suspected and researched.

However, specific recommendations for the diagnosis of PAD in diabetic patients are not yet available in the literature. It is always suggested to perform a clinical examination and then use non-invasive diagnostic tests to identify PAD. However, these tests do not seem to be able to exclude the disease. The multiple non-invasive tests approach recently proposed (with ABI, TBI, CWD, and TcPO2) is presumably an effective strategy in diabetic patients. However, if the clinical suspicion is strong, it may be appropriate to resort to imaging methods. Even more, the diagnosis of PAD is crucial in DFD, as is the stratification of the risk of amputation to identify patients who are candidates for revascularization. The WIfI classification seems to be suitable for this condition.

It is hoped that, in the future, there will be greater interest in and further studies on this topic to provide stronger recommendations to clinicians for an earlier diagnosis of the disease.

## Figures and Tables

**Table 1:** Leriche Fontaine and Rutherford PAD classification.

Leriche Fontaine Classification [20]				Rutherford Classification [21]		
*Grade*		*Symptomatology*		*Grade*	*Category*	*Symptomatology*
I		Asymptomatic	⇔	0	0	Asymptomatic
II	II a	Mild claudication	⇔	I	1	Mild claudication
				I	2	Moderate claudication
	II b	Moderate to severe claudication		I	3	Severe claudication
III		Ischemic pain at rest	⇔	II	4	Ischemic pain at rest
IV		Ulceration/gangrene	⇔	III	5	Tissue loss (mild)
				III	6	Tissue loss (major)

**Table 2:** Cut-off values of hemodynamic parameters for diagnosis of CLI or reduced probability of wound healing reported in main guidelines.

	Hemodynamic Parameters for Diagnosis of CLI or Reduced Probability of Wound Healing			
	AP	ABI	TP	TcPO2
Consensus EWG I [26]	≤50 mmHg			
Consensus EWG II [27]	≤50 mmHg		≤30 mmHg	
Rutherford Classification [21]	<40 mmHg for class 4		<30 mmHg for class 4	
	<60 mmHg for class 5 and 6		<40 mmHg for class 5 and 6	
TASC I [28]	<50–70 mmHg		≤30–50 mmHg	≤30–50 mmHg
TASC II [19]	<50 mmHg, if rest pain <70 mmHg, if wounds or gangrene		<30 mmHg, if rest pain <50 mmHg, if wounds or gangrene	≤30–50 mmHg
WIfI classification [29]	70–100 mmHg (grade 1 ischemia)	0.60–0.79 (grade 1 ischemia)	40–59 mmHg (grade 1 ischemia)	40–59 mmHg (grade 1 ischemia)
	50–70 mmHg (grade 2 ischemia)	0.40–0.59 (grade 2 ischemia)	30–39 mmHg (grade 2 ischemia)	30–39 mmHg (grade 2 ischemia)
	<50 mmHg (grade 3 ischemia)	<0.40 (grade 3 ischemia)	<30 mmHg (grade 3 ischemia)	<30 mmHg (grade 3 ischemia)
2016 AHA/ACC PAD Guidelines [72]			<30 mmHg	<30 mmHg
2017 ESC PAD Guidelines [15]	<50 mmHg	<0.40	<30 mmHg	<30 mmHg
2019 Global vascular guidelines on the management of CLTI [35]			<30 mmHg	<30 mmHg
2019 ESVM PAD Guidelines [7]			<30 mmHg	<30 mmHg
2023 IWGDF, ESVS, SVS PAD Guidelines in Diabetes Mellitus and a Foot Ulcer [17]	<50 mmHg	<0.40	<30 mmHg	<30 mmHg
2024 ESVS PAD Guidelines [18]			<30 mmHg	<30 mmHg
2024 ACC/AHA PAD Guidelines [16]			<30 mmHg	<30 mmHg
